# Crack/cocaine use to manage xylazine exposure in fentanyl in Connecticut: findings from a convergent mixed methods study

**DOI:** 10.1186/s12954-026-01459-1

**Published:** 2026-05-02

**Authors:** Katherine Hill, Emma T. Biegacki, Gabrielle Bogut, Elizabeth Znamierowski, Cameron Breen, River Rose, Corona Zhang, Lauretta E. Grau, Jenna L. Butner, Robert Heimer, Kimberly L. Sue

**Affiliations:** 1 Department of Epidemiology of Microbial Diseases, Yale School of Public Health, New Haven, CT USA; 2 Yale Program in Addiction Medicine, Yale School of Medicine, New Haven, CT USA; 3 Department of General Internal Medicine, Yale School of Medicine, New Haven, CT USA; 4 New Haven Syringe Services Program, New Haven, CT USA; 5 Infectious Diseases, Internal Medicine, Yale School of Medicine, New Haven, CT USA; 6 Liberation Programs, Bridgeport, CT USA; 7 Connecticut Harm Reduction Alliance, Hartford, CT USA; 8 Alliance for Living, New London, CT USA

**Keywords:** Harm reduction, Xylazine, Fentanyl, Crack, Cocaine, Stimulants

## Abstract

**Background:**

In the context of the fourth wave of the opioid overdose crisis, defined by fentanyl and stimulant co-use, we aimed to explore if there might be a relationship between exposure to xylazine in the fentanyl supply and patterns of crack/cocaine use.

**Methods:**

This mixed methods study used data from four Connecticut-based sources: (1) the Office of the Chief Medical Examiner for fatal overdoses, 2019–2023, (2) community-based drug checking data, 2023–2024, (3) a structured survey of people who use drugs, 2024, and (4) one-on-one in-depth interviews, 2024.

**Results:**

Xylazine was detected in 19.2% of overdose fatalities involving fentanyl or a fentanyl analog (*n* = 5,849). Those with xylazine detected had a higher proportion of cocaine simultaneously detected, though this was not statistically significant (54.0% vs. 51.1%, *p* = 0.085). Cocaine detection rose over time in the fatality data, regardless of xylazine detection. There were 1,048 drug samples submitted to harm reduction organizations for drug checking. Using Fourier Transform Infrared Spectroscopy, 573 of the samples submitted tested positive for fentanyl or a fentanyl analog; 45.2% of these also tested positive for xylazine. Of 1048 samples, 276 tested positive for cocaine. Among the survey sample (*n* = 88), 62.5% of participants self-reported xylazine exposure. Those who reported lifetime xylazine exposure also reported higher past-year crack/cocaine use, though this was not statistically significant (89.1% vs. 81.8%, *p* = 0.336). Among our interview sample (*n* = 31), three themes emerged: (1) participants felt there was an inevitability of polysubstance use due to an increasingly volatile drug supply, (2) participants increased their crack/cocaine use in response to perceived exposure to xylazine in their fentanyl supply, and (3) participants’ drug patterning changed due to xylazine’s presence (e.g., timing, drug sequencing, etc.). Two meta-inferences were identified: (1) overdose fatality data likely underestimate the “true” prevalence of xylazine in the street supply, and (2) people who use drugs are using crack/cocaine in response to xylazine in their local fentanyl supply.

**Conclusions:**

Xylazine exposure has likely contributed to an adaptive and increasing use of crack/cocaine among people who use drugs in Connecticut. It is imperative that practitioners and policymakers are prepared to manage this triad of opioid-stimulant-sedative polysubstance use.

## Background

Overdose deaths over the past decade have largely been attributed to the rise of fentanyl in the unregulated drug supply. More recently, increasing co-use of fentanyl and stimulants—most notably methamphetamine and crack/cocaine—has been detected in nearly half of all overdose deaths in the United States (US) from January 2021 to June 2024 [1–3]. Such polysubstance use, defined as the use of multiple psychoactive substances—intentionally or unintentionally, either simultaneously or concurrently in a short period of time—is increasingly common [4–7]. Further, given the complexity of the unregulated drug supply in the US, people who use drugs (PWUD) often engage in polysubstance use unknowingly as they are inadvertently exposed to various substances and adulterants in their drug(s) of choice [8, 9]. Conversely, some PWUD may intentionally use multiple drugs for a variety of reasons. Thus, polysubstance use is now the norm, not the exception [10, 11].

Polysubstance use increasingly includes veterinary drugs such as medetomidine and xylazine [12, 13]. Xylazine, an α-2 adrenergic receptor agonist never approved by the Food and Drug Administration for use in humans is found almost exclusively in unregulated fentanyl [12, 14–16]. Few case reports document xylazine exposure alone (i.e., without fentanyl) in humans; those that exist typically describe xylazine-related sequelae in individuals working closely with animals (e.g., veterinarians, farmers) rather than among PWUD [17–20]. Fentanyl and xylazine co-use in humans has led to adverse effects (deemed xylazine-related outcomes or XROs), which include, but are not limited to, intense sedation, overdose, withdrawal, and non-infectious wounds that are slow to heal [15, 21–27].

Having a nuanced understanding of polysubstance use is crucial [5]; understanding use behaviors, motivations, patterns of PWUD, alongside any drug interactions, and how these use patterns shape overdose vulnerability are crucial when building better interventions in both harm reduction and clinical practice that could decrease rates of overdose and other drug-related harms. To our knowledge, no studies to date have explored the use patterns and health impact of the addition of stimulants to fentanyl and xylazine co-use. Thus, our research study employed a mixed methods approach to better understand the polysubstance use patterns in Connecticut (CT), US. The state has exhibited features emblematic of the changing drug marketplace; it was among the first to note the dominance of fentanyl in street drugs (2014), the growing presence of xylazine (2019) and the increase in the presence of cocaine in fatal opioid-detected overdoses over the last few years [28–31]. In this report, we explore the increase in crack/cocaine in combination with fentanyl and xylazine from four angles: fatal overdose data, community-based drug checking, quantitative survey data, and open-ended interviews with PWUD. We have chosen to limit our considerations of methamphetamine and other amphetamine-type substances herein, as our previous research and work in the field indicates their use among PWUD in Connecticut has long been uncommon [32–34].

## Methods

**Fig. 1:**
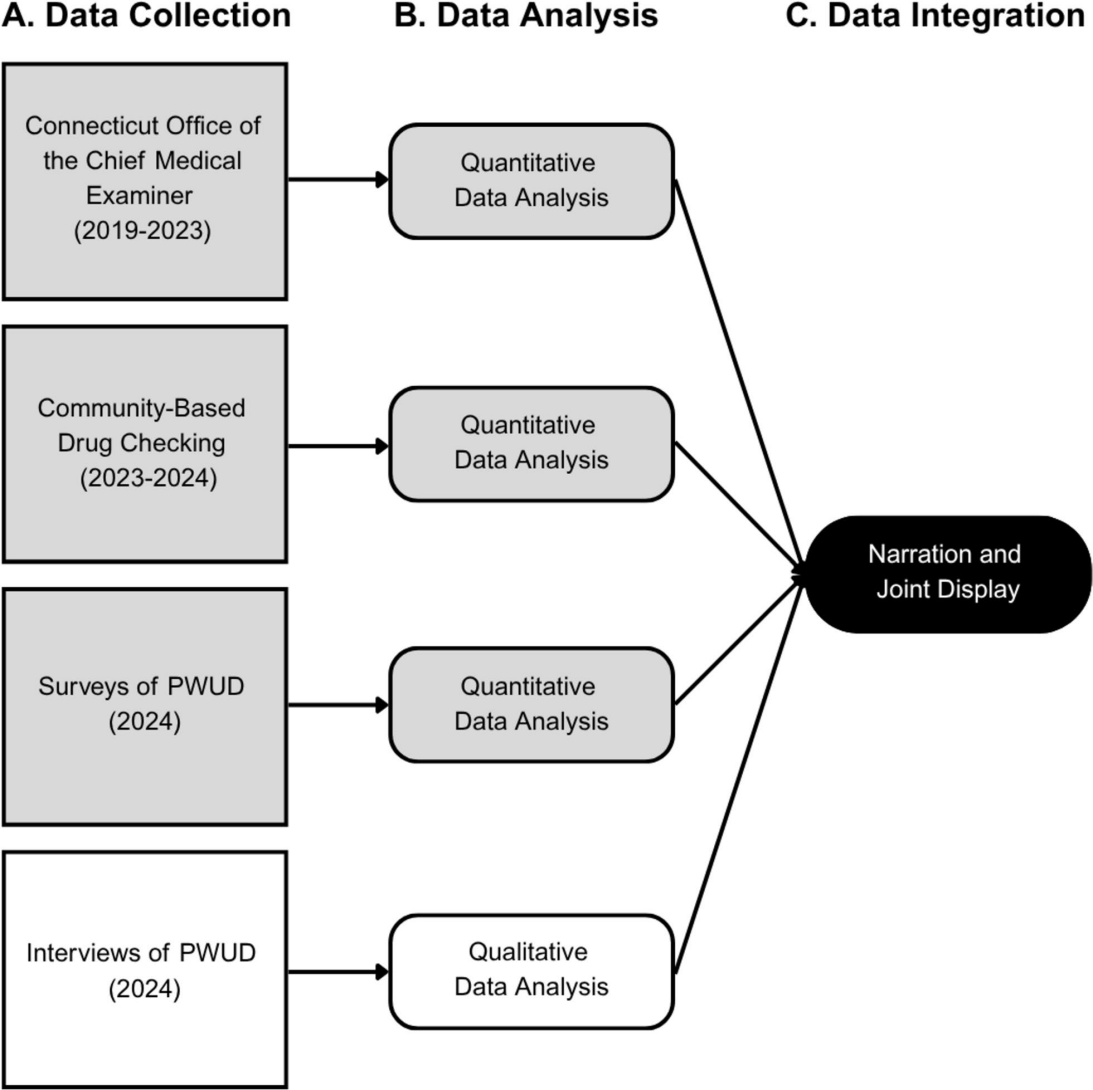
Convergent mixed methods study design. **A** Data Collection. There were four data sources used for this study design. Gray boxes indicate quantitative data sources while white boxes indicate qualitative data sources, **B** Data Analysis. Both quantitative and qualitative analysis was used, based on the data source, **C** Data Integration. Narration and joint display techniques were used to integrate the four data sources to form meta-inferences

To better understand the phenomenon of stimulant use with fentanyl and xylazine, we used a convergent mixed methods design to analyze four data sources (Fig. 1**)**. Brief summaries of the data collection and data analysis for each data source are described below, as well as the integration strategy employed.

## Connecticut Office of the Chief Medical Examiner (OCME) data (2019–2023)

### Data collection

The Connecticut Office of the Chief Medical Examiner (OCME) granted our team access to its database of all deaths from which we extracted all opioid-detected accidental or undetermined opioid-detected overdose deaths from 2019 to 2023. Opioid-detected deaths were defined as an unintentional death with a quantifiable amount of opioid in the toxicology report or an opioid listed in the cause of death (when toxicology reports were unavailable). All data were validated by reviewing source documents that included (1) date of death, (2) demographic information including sex (male/female), race/ethnicity (White, Black, Hispanic, Other race/ethnicity, or Unknown), and age (year) at death, (3) cause and manner of death and toxicological test results, if available, and (4) the addresses of the decedent’s residence, injury, and/or death, if available. Toxicological test results cannot distinguish between crack and cocaine; thus, OCME data will refer to all related findings as just “cocaine.”

### Data analysis

For this study, we further restricted the dataset to those cases involving fentanyl or its analogs, as xylazine has been found to be almost exclusively in the fentanyl supply (12). We also created an indicator variable for xylazine positivity based on if xylazine was (a) present in a quantifiable amount in the toxicological report or (b) in the cause of death when there was no toxicological report. We subsequently dichotomized the dataset based on presence or absence of xylazine. We then used basic summary statistics of sociodemographic variables and toxicological report findings (i.e., fentanyl, xylazine, cocaine, and amphetamine detection) for the total sample, as well as for decedents with and without xylazine in their systems at the time of death. Bivariate statistics were calculated using chi-square tests for categorical variables and t-tests for continuous variables. Spatial data were analyzed at the county level using the injury location. If the county of injury was unavailable, we defaulted to the county of residence and if also unavailable, to the county where the death occurred.

### Community-based drug checking data (2023–2024)

#### Data collection

The research team collaborated with four CT-based harm reduction organizations [Liberation Programs (LP), Connecticut Harm Reduction Alliance (CTHRA), Alliance for Living (AFL), and the New Haven Syringe Service Program (NHSSP)] serving non-overlapping regions of CT. These programs offered drug checking services and used in-house real-time testing: (1) rapid single-use immunoassay test strips to detect fentanyl or xylazine (BTNX Inc., Rapid Response™, Pickering, Ontario, CA) and (2) point of care Fourier Transform Infrared Spectroscopy (FTIR) (Bruker ALPHA II, Billerica, Massachusetts, US) [35, 36]. Programs could send samples for further testing to the Connecticut Public Health Laboratory for confirmatory gas chromatography–mass spectrometry or liquid chromatography-quadrupole time-of-flight spectroscopy (collectively abbreviated GCMS/LC-qTOF) [37]. Clients of the programs could submit drug remnants (e.g., wax baggies, wrappers, pills) as a suspected substance for analysis when a sufficient amount of sample is available. The programs provided our study team with drug checking data from samples submitted voluntarily by PWUD who used their services for 2023 and 2024. No demographic or other identifying information was collected from the PWUD who submitted the samples.

#### Data analysis

We tabulated the frequency of xylazine and cocaine in samples submitted as fentanyl, as well as the frequency of xylazine and fentanyl in samples submitted as cocaine, using all methodologies whenever possible (i.e., test strips, FTIR, and GCMS/LC-qTOF). We report the presence of any congener of fentanyl in the aggregate, as fentanyl test strips cannot distinguish among them and each product has different sensitivities in terms of amount and congener recognition [38, 39].

Further, analysis of FTIR and GCMS/LC-qTOF spectroscopy profiles allowed determination of the relative concentration of fentanyl, xylazine, and cocaine in the submitted samples. The community-based drug sites reported relative concentration estimates of detected components based on the limitations of the analytical instrument used and the limitations of the sample itself. For GCMS/LC-qTOF, component designations were categorized as *major* (30–100%), *minor* (10–29%), or *trace* (< 10%) compared against the most abundant substance in the sample [40]. These thresholds provided a standardized framework for reporting relative abundance across sites. In contrast, FTIR concentration estimates are less precise, with FTIR component designations closely mirroring those of GCMS/LC-qTOF [*major* (30–100%), *minor* (10–29%), or *trace* (< 10–5%)] with several important distinctions. The designations of major, minor or trace are technician-dependent and will reflect their best estimate of the substance’s composition. Designation of “unknown” reflects an FTIR technician's uncertainty in the presence of a specific substance in the sample.

We additionally explored if there was a temporal trend in xylazine positivity among fentanyl samples; using R software we used linear regression analysis fit to monthly variations in xylazine prevalence over a 24-month period.

### Structured surveys of PWUD (2024)

#### Data collection

During 2024, our study team—in collaboration with seven Connecticut harm reduction-oriented organizations (LP, CTHRA, AFL, NHSSP, McCall Behavioral Health Network, Middletown Harm Reduction Initiative, and The Village for Families & Children)—recruited a convenience sample of PWUD aged ≥ 18 who reported drug use in the prior 6 months to assess their perceptions about and experiences with xylazine. Our 10-min survey covered topics such as substance use history and perceived exposure to xylazine; this manuscript will cover the portions of the survey that relate to participant demographics (including county of residence, age, race, ethnicity, gender), drug use in the last year, and self-reported exposure to xylazine. Exposure to xylazine was assessed by the close-ended question “Have you ever used xylazine or drugs that contain xylazine?”; if participants selected “Yes—I have used xylazine intentionally” or “Yes—I have used xylazine but by accident,” they were dichotomized into the xylazine exposed group, if they selected “No,” or “I am not sure,” they were placed into the unexposed group. Participants were compensated $10 for completing the survey. More detailed information on the methods (i.e., sampling, topic guide development, etc.) can be found elsewhere [32].

#### Data analysis

Based on responses to half-year substance use questions, data were dichotomized by self-reported fentanyl use with or without exposure to xylazine. Basic summary statistics were then used to describe the study sample by sociodemographic and other drug use characteristics. Bivariate statistics were used to compare the xylazine exposure groups; a chi-square test or Fisher Exact (depending on sample size) was used for categorical variables and a t-test was used for continuous variables.

### In-depth interviews of PWUD (2024)

#### Data collection

Using purposive sampling—based on the axes of site location and self-reported xylazine exposure—our study team conducted one-on-one interviews with a subset of survey participants selected from among those who indicated that they were interested in participating in follow-up interviews. No additional eligibility criteria were imposed. Interviews were conducted using a semi-structured interview guide that covered topics such as awareness of xylazine, impact of xylazine on drug use, and XROs (i.e., wounds, sedation, withdrawal, overdose). Participants were compensated an additional $30 for completing an interview that lasted approximately 35 min. All interviews were audio-recorded, transcribed, and cleaned by the study team. Recruitment continued until theoretical saturation was reached [41]. More detailed information on the methods used in these interviews can be found elsewhere [32].

#### Data analysis

A codebook was inductively created and iteratively refined by three members of the research team who are trained harm reduction researchers (K.H., E.T.B., and G.B.). E.T.B. and G.B. double coded all transcripts using NVivo software, version 12 (QSR International) and met frequently to reconcile discrepancies in their coding process. Our team (K.H., E.T.B., and G.B.) met to discuss and elucidate themes using thematic analysis techniques, [42] with a focus on the ways in which PWUD were navigating exposure to xylazine through polysubstance use.

### Data integration

We pursued data integration as a strategy to increase potential validity and generalizability of our findings. Integration of the data was conducted with the goal of establishing whether each data source told the same story in addressing the question of “What is the association between exposure to xylazine in the fentanyl supply and patterns of cocaine use?” Each dataset provided a different analytic lens:

- The OCME data allowed us to explore the prevalence of xylazine exposure amongst those whose deaths in Connecticut involved fentanyl. Additionally, it also allowed us to explore the potential patterning of crack/cocaine use among PWUD who used fentanyl with xylazine.
- The community-based drug checking data allowed us to explore xylazine’s prevalence in our state drug supply, alongside that of cocaine and fentanyl, as reflected in the overall sample of drug remnants. This sample provides an entirely different, but complementary, assessment from the OCME data insofar as it provides an estimate of what (non-deceased) PWUD are actively consuming.
- Our survey of PWUD allowed us to explore the prevalence of xylazine exposure as well as potential patterning of crack/cocaine use among PWUD.
- Lastly, our interview data allowed our study team to contextualize and understand the diversity of human experience behind the three quantitative datasets we analyzed. Additionally, it allowed us to hypothesize directionality and temporality of certain findings, especially in the context of the cross-sectionality of our quantitative data sources.

We utilized both narration and joint display data integration techniques to merge analyzed results to form meta-inferences [43–45].

## Results

### Connecticut OCME data (2019–2023)

Table 1 provides an overview of the OCME data, wherein 5849 people experienced a fatal overdose involving fentanyl or a fentanyl analog. Xylazine was present in 19.2% of these fatalities on average annually, although the proportion peaked in 2022 when xylazine was identified in 27.2% (341/1252) of fentanyl-related deaths.

**Table 1:** Temporal, sociodemographic, and postmortem toxicological report characteristics of decedents with a fentanyl-detected overdose, stratified by detection of xylazine, connecticut office of the chief medical examiner (2019–2023)

Characteristic	All fentanyl-detected overdose (*N* = 5849)		Fentanyl-detected overdose with xylazine detected (*n* = 1125)		Fentanyl-detected overdose without xylazine detected (*n* = 4724)		
	N	%	n	%	n	%	*P* value
Year							** < 0.001**
2019	978	16.72	73	6.49	905	19.16	
2020	1163	19.88	139	12.36	1024	21.68	
2021	1319	22.55	290	25.78	1029	21.78	
2022	1252	21.41	341	30.31	911	19.28	
2023	1137	19.44	282	25.07	855	18.10	
County							** < 0.001**
Fairfield	922	15.76	115	10.22	807	17.08	
Hartford	1739	29.73	447	39.73	1292	27.35	
Litchfield	259	4.43	36	3.20	223	4.72	
Middlesex	178	3.04	34	3.02	144	3.05	
New Haven	1926	32.93	259	23.02	1667	35.29	
New London	507	8.67	135	12.00	372	7.87	
Tolland	123	2.10	30	2.67	93	1.97	
Windham	195	3.33	69	6.13	126	2.67	
Age (Mean, SD)	44.70	12.67	44.96	12.49	44.63	12.71	0.442
Race/ethnicity							**0.001**
White	3720	63.69	756	67.26	2964	62.84	
Black	974	16.68	141	12.54	833	17.66	
Hispanic	1059	18.13	211	18.77	848	17.98	
Other	51	0.87	11	0.98	40	0.85	
Unknown	37	0.63	5	0.44	32	0.68	
Sex							0.486
Male	4465	76.42	850	75.62	3615	76.61	
Female	1378	23.58	274	24.38	1104	23.39	
*Other substances detected*							
Cocaine (*n* = 5808)	3000	51.65	607	53.96	2393	51.10	0.085
2019	441	45.14	36	49.32	405	44.80	
2020	523	45.13	66	47.48	457	44.80	
2021	666	51.00	160	55.17	506	49.80	
2022	391	55.73	190	55.72	501	55.73	
2023	679	60.30	155	54.96	524	62.09	
Amphetamines ^a^ (*n* = 5776)	328	5.68	61	5.42	267	5.74	0.679s
2019	56	5.74	2	2.74	54	5.98	
2020	74	6.40	10	7.19	64	6.29	
2021	82	6.32	25	8.62	57	5.66	
2022	62	5.02	15	4.40	47	5.26	
2023	54	4.86	9	3.19	45	5.42	

^a^Includes other stimulants as well, such as eutylone, MDMA, N,N-diMe-pentylone, MDA

When looking at the decedents’ sociodemographic characteristics, the average age of those experiencing a fentanyl-detected overdose was 44.7 years (SD = 12.7), and the majority was male (*n* = 4465, 76.4%). Statistically significant differences existed when comparing race/ethnicity (*p* = 0.001); among all fentanyl-involved overdose fatalities, there were proportionally more White decedents who had xylazine detected upon death (*n* = 756, 67.3%) than those without xylazine (*n* = 2964, 62.8%). Additionally, there were proportionally less Black decedents with xylazine detected (*n* = 141, 12.5%) than those without xylazine (*n* = 833, 17.7%). At the county level throughout Connecticut, Hartford (*n* = 447) and New Haven (*n* = 259) had the highest prevalence of decedents in whom both fentanyl and xylazine were detected; countywide, differences in xylazine detection were significantly different (*p* < 0.001).

There were non-statistically significant findings regarding cocaine in the toxicology reports or listed in the cause of death when comparing those with and without xylazine detected. Those with xylazine detected had a higher proportion of cocaine also detected (54.0% vs. 51.1%, *p* = 0.085); however, cocaine detection rose over time (2019–2013) in both those with and without xylazine detection (from 49.3% to 55.0%, and from 44.8% to 62.1%, respectively).

### Community-based drug checking data (2023–2024)

**Fig. 2:**
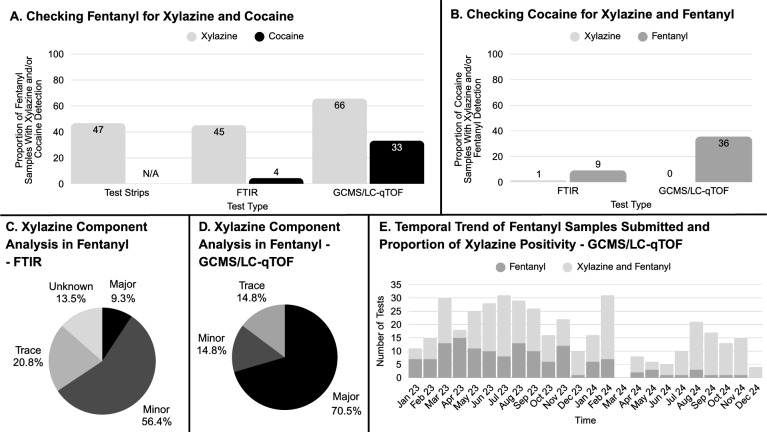
Aggregate findings from drug trash and remnant donation samples from three community-based drug checking programs (2023–2024), **A** Proportion of xylazine and cocaine in fentanyl samples. Regarding the three test types, the overall sample sizes were as follows: test strips: *n* = 671; FTIR: *n* = 573; GCMS/LC-qTOF: *n* = 410, **B** Proportion of xylazine and fentanyl in cocaine samples. Regarding the two test types, the overall sample sizes were as follows: FTIR: *n* = 276; GCMS/LC-qTOF: *n* = 381, **C** Component analysis of the xylazine found in fentanyl samples using FTIR. This analysis was based on *n* = 259 samples that tested positive for xylazine using FTIR, **D** Component analysis of the xylazine found in fentanyl samples using GCMS/ LC-qTOF, This analysis was based on *n* = 271 samples that tested positive for xylazine using GCMS or LC-qTOF, **E** Proportion of xylazine positivity in fentanyl samples from 2023–2024, by month, using GCMS/ LC-qTOF. Abbreviations: FTIR: Fourier transform infrared spectroscopy; GCMS: gas chromatography-mass spectrometry; LC-qTOF: Liquid chromatography-quadrupole time-of-flight

Trends in presence of the co-occurrence of fentanyl, xylazine, cocaine in samples tested by the different methods (test strips, FTIR, GCMS/LC-qTOF) at the four community-based drug checking programs can be found in Fig. 2. The four community-based drug checking programs received 1280 voluntary samples for checking during the study period. However, only 1101 samples were tested with fentanyl test strips; 671 tested positive for fentanyl or a fentanyl analog (Fig. 2, panel A). Of these, 314 (46.8%) tested positive for xylazine using a xylazine test strip at 5 mL dilution. Depending on sample quantity, and program capacity, not all samples were tested further with FTIR and/or GCMS/LC-qTOF.

### FTIR and GCMS/LC-qTOF—samples of fentanyl

Using FTIR, 1048 samples were tested (Fig. 2, panel A); 573 (54.7%) samples tested positive for fentanyl or a fentanyl analog. Of these samples, 259 (45.2%) tested positive for xylazine and 25 (4.4%) tested positive for cocaine. A total of 748 samples were tested using GCMS/LC-qTOF, and 410 (54.8%) tested positive for fentanyl or a fentanyl analog. Of these samples, 271 (66.1%) tested positive for xylazine and 137 (33.4%) tested positive for cocaine.

Figure 2, panel C provides the component analysis of the 259 fentanyl samples that tested positive for xylazine using FTIR; xylazine was typically a minor component (56.4%). Of the *n* = 271 fentanyl samples that tested positive for xylazine using GCMS/LC-qTOF, the component analysis suggested that fentanyl was most often a major component (70.5%, Fig. 2, panel D).

### FTIR and GCMS/LC-qTOF—samples of cocaine

Of the 1,048 samples that were tested by FTIR, 276 (26.3%) samples tested positive for cocaine (Fig. 2, panel B). Only 4 (1.5%) of these cocaine samples tested positive for xylazine (primarily in minor amounts, 50.0%) while *n* = 25 (9.1%) tested positive for fentanyl or a fentanyl analog (mostly in minor amounts, 52.0%). Further testing of *n* = 748 samples using GCMS/LC-qTOF detected *n* = 381 samples which tested positive for cocaine. Among these, none tested positive for xylazine, while *n* = 137 (36.0%) tested positive for fentanyl or a fentanyl analog (mostly in major amounts, 67.2%).

Using the FTIR method, cocaine was detected in 4.4% of the fentanyl samples; when cocaine was found, it was predominantly a major component (60.0% = “major”). When using the GCMS/LC-qTOF method, cocaine was detected in 33.4% of the fentanyl samples; when cocaine was identified by GCMS/LC-qTOF, it was most often in trace components (40.2% = “trace”).

### Temporal trends

Over a two-year period (2023–2024), there were monthly variations in the number of samples provided to the programs and the proportion testing positive for xylazine (Fig. 2**, panel E**). Early 2023 saw lower xylazine positivity per GCMS/LC-qTOF tests per month, while late 2024 saw higher xylazine positivity (R^2^ = 0.373, *p* = 0.002), although fewer samples were being tested.

### Structured surveys of PWUD (2024)

Characteristics of those who participated in the xylazine-focused surveys are summarized in Table 2. Overall, 88 people who reported consuming fentanyl in the last year participated in this survey, with 62.5% (*n* = 55) reporting xylazine exposure. Those reporting xylazine exposure in their fentanyl were younger than those without such reported exposure (45.1 vs. 52.9 years, *p* = 0.001). Of the four other drugs we inquired about, only use of benzodiazepines in the past year was associated with xylazine exposure (60% vs. 33% for those not exposed, *p* = 0.015). There were no other statistically significant differences between those reporting fentanyl use with or without xylazine exposure.

**Table 2:** Sociodemographic and substance use related characteristics people who use fentanyl, stratified by reported exposure to xylazine, survey in connecticut (2024)

Characteristics	Total (*N* = 88)		Fentanyl use and reported xylazine exposure (*n* = 55)		Fentanyl use and no reported xylazine exposure (*n* = 33)		p-value
	N	%	n	%	n	%	
County of residence							0.082
Fairfield	23	26.14	10	18.18	13	39.39	
New Haven	21	23.86	16	29.09	5	15.15	
Hartford	20	22.73	11	20.00	9	27.27	
New London	15	17.05	12	21.82	3	9.09	
Middlesex	6	6.82	3	5.45	3	9.09	
Litchfield	3	3.41	3	5.45	0	0.00	
Age (*n* = 86), (Mean, SD)	48.05	11.40	45.06	11.50	52.85	9.56	**0.001**
Race*							
White	65	73.86	33	60.00	15	45.45	0.185
Black or African American	48	54.55	13	23.64	10	30.30	0.491
Native American/Alaskan Native	1	1.14	1	1.82	0	0.00	0.436
Other race	16	18.18	9	16.36	7	21.21	0.568
Ethnicity							0.130
Hispanic or Latino	26	29.89	13	24.07	13	39.39	
Not Hispanic or Latino	61	70.11	41	75.93	20	60.61	
Gender							0.676
Male	61	69.32	39	70.91	22	66.67	
Female	27	30.68	16	29.09	11	33.33	
Self-reported drug use in last year*							
Benzodiazepines	44	50.00	33	60.00	11	33.33	**0.015**
Crack/cocaine	76	86.36	49	89.09	27	81.82	0.336
Amphetamines**	16	18.18	12	21.82	4	12.12	0.393

*Participants could select multiple categories, so columns may not sum to total or 100%

**Includes methamphetamines and/or other forms of speed (did not include pharmaceutical medication)

### In-depth interviews of PWUD (2024)

Among the 31 participants who were interviewed, substantial polysubstance use was reported; in the last year, 87.1% (*n* = 27) reported using fentanyl, 80.6% (*n* = 25) reported using crack cocaine, and 58.1% (*n* = 18) reported using powder cocaine. Overall, *n* = 27 (87.1%) reported xylazine exposure in their lifetime. The opioid-stimulant-sedative triad of interest was common amongst interview participants, as 74.2% (*n* = 23) reported past year use of fentanyl, crack and/or powder cocaine, and exposure to xylazine. Of note, all participants who used fentanyl and crack/cocaine in the last year reported exposure to xylazine. Thematic analysis from these interviews revealed three main topics related to polysubstance use (1) perception, (2) motivators, and (3) patterning. Participants below have been randomly assigned aliases.

#### Theme 1: Most participants perceived that polysubstance use was likely inevitable in today’s drug supply.

Many interview participants who reported both fentanyl and crack/cocaine use endorsed a sense of inevitability regarding their exposure to xylazine, due to (a) the perceived ubiquity of fentanyl in the unregulated opioid supply, (b) the growing prevalence of xylazine in the fentanyl supply, and/or (c) the potential for fentanyl contamination in the crack/cocaine or other stimulant supply.

While it may not be everyone’s preference to use fentanyl, xylazine, and crack/cocaine, many participants have accepted polysubstance use as the default within the broader context of a fentanyl-saturated market in which xylazine may be present in unknown quantities. For instance, Martin, a 40-year-old White male who has injected opioids since he was 19, stated:

*“*It’s gotta be [xylazine], because…they’re putting the fentanyl in everything, and I think it’s [i.e., xylazine] in all this fentanyl*.”*

Other participants generally agreed that fentanyl was the primary vehicle for xylazine exposure, given the ubiquity of fentanyl in the unregulated supply, but their perceptions of exactly how often or how much xylazine is present in the unregulated fentanyl varied, with participants offering estimates ranging from 10 to 90%. Ultimately, uncertainties about whether xylazine may be present in one’s drug of choice prompted many participants to use drug checking services or express a growing curiosity about such services. For example, Veronica, a 60-year-old Hispanic woman who first witnessed xylazine use and the impact of XROs in her native Puerto Rico, stated:

*“*It’s [i.e., drug checking’s] good, because then you know exactly what you're putting in your body*.”*

Veronica, and other participants, recognized that drug testing services can provide PWUD more information about what drugs they are using, and whether they may be exposed to xylazine.

A few participants had concerns about polysubstance use and exposure to drugs they did not intend to use. For example, Derek, a 55-year-old Black man who first began using crack cocaine in the 1980s, described an incident involving a pipe borrowed from someone who did not disclose that it had recently been used to smoke fentanyl. He stated,

*“*[I] borrowed a…[crack] pipe one day – ‘cause that's how people on the street be – borrowed a fucking pipe. Now you could say, ‘that is some junk man. There is some fentanyl on top of here.’ But they let me hit it…I was like, ‘What the fuck is wrong? I only smoked a dime!’… But he just let me stick my rock in there. And you know, now I’m affected.*”*

Derek believed, in this instance, he was inadvertently exposed to xylazine from fentanyl smoked in the pipe before he used it to smoke crack. Thus, he felt as though he engaged in polysubstance use unintentionally. It was not until he experienced the effects afterwards—as they were different from what he was expecting given his familiarity with prior experiences of crack use—that he worried about the potential contents of the pipe. Overall, most participants were aware of the presence of xylazine in the unregulated drug supply and of resources available to increase knowledge about exposure risks and reduce related harms. Participants’ feeling that ‘polysubstance use may be unavoidable’, coupled with their uncertainty about xylazine’s true prevalence, appeared to shape engagement with and interest in polysubstance use, which is further explored in subsequent themes.

#### Theme 2: Many participants indicated that they were motivated to use crack/cocaine when exposed to xylazine in their fentanyl supply, though the motivations were varied

Many participants reported an increase in the frequency or amount of their crack/cocaine use in response to xylazine exposure in their fentanyl. For instance, Heather, a 44-year-old White woman, described that,

*“*They [i.e., crack and fentanyl with xylazine] go hand in hand. I do one with the other, usually not one without the other...Always down before you go up.*”*

Yet, the key motivators for using crack/cocaine varied across participants: to balance out the sedative effects of xylazine, to improve their level of functioning and safety, and to achieve perceived harm reduction benefits (i.e., related to overdose response). Each of these motivating factors will be described in more detail below.

Many participants described crack/cocaine (i.e., an “upper”) as a tool for achieving balance while using fentanyl and xylazine (i.e., “downers”), in that crack/cocaine helped participants feel more alert, calm, and well-regulated, and served to mitigate the intensely embodied effects of the opioid and sedative. For instance, Heather continued to describe this succinctly,

*“*It [i.e., crack cocaine] just pushes, so you're not so noddy you can't function. When I’m on drugs I can still function. [Crack cocaine] makes it so you can.*”*

In this way, participants, like Heather, reported that including crack/cocaine in their substance use routines allowed them to go about their daily lives without feeling overly sedated. In fact, this phenomenon of crack/cocaine helping improve functioning was commonly described. Charlie, a 55-year-old Black man, explained,

*"*[If I used fentanyl and xylazine,] I won't be able to sit here and conversate or talk or walk if I don't do the crack. If I just do the xylazine, maybe shoot it, I'm antisocial! I can't function. I'm out somewhere. Done. That's why I make sure I get crack. And when I smoke a crack, I can walk around, I can talk, I'm functional. But without the crack? I'm not functional.*”*

Thus, there is an emerging functional utility for crack/cocaine consumption in the context of xylazine. To the extent that this combination of drugs allowed participants to maintain or regain basic functioning in their daily lives, it facilitated their ability to work, engage in relationships, and fulfill responsibilities.

Beyond preservation of daily functioning, participants also described using crack/cocaine in conjunction with xylazine in fentanyl in order to increase their sense of safety. For instance, Charlie further recounted an experience where he struck a pole while driving under the influence of what he believed was fentanyl with xylazine. This event motivated him to begin using crack/cocaine whenever he used fentanyl with xylazine:

*“*Scared the hell out of me. I could have been dead. That's why I won't do that [i.e., fentanyl with xylazine] without crack… It puts a horse or an elephant down. What do you think it is going to do to a human? [The crash] scared the hell out of me. That's why I make sure I got crack every time.*”*

For participants like Charlie, adding crack/cocaine to their daily drug-use routine has become a way to ensure their own safety. Without doing so, these participants faced the risk of becoming over-sedated from the xylazine when carrying out daily activities that could put themselves or others at risk of significant harm.

Another reason some participants endorsed using crack/cocaine when exposed to fentanyl with xylazine was its perceived ability to prevent “nodding out” or even attempt to counter opioid overdoses with xylazine involved. Cynthia, a 50-year-old woman, explained that she often would take crack when she was exposed to a “sedating fent”:

*“*So, the thought is that crack cuts the life of the dope. The more crack you do, the thought is that the dope will keep decreasing [in its effect]. You keep smoking crack to wake up and come back. It's worked on me... [The people I was smoking with were] like, ‘here, take a hit, take a hit.’ They kept making me take hits [because] I was leaving [i.e., nodding/falling out].*"*

In other words, participants reported using crack/cocaine alongside fentanyl with xylazine in order to counteract the opioid and sedating effects, and thus helping them to “come back” (i.e., stay alive) or stop “falling out” (i.e., stay awake). For many participants who reported a fear of excessive sedation, crack/cocaine was perceived as a harm reduction or preventative measure.

#### Theme 3: Participants described how they patterned their use of fentanyl, xylazine, and crack/cocaine based on a number of unique factors.

While many participants described initiating or increasing their crack/cocaine use in response to the potential presence of xylazine in their drug(s), primarily fentanyl, how they chose to sequence their use of multiple drugs varied. Martin explained,

*“*[I]t’s [i.e., xylazine’s] making a lot of people do cocaine, because you're getting knocked out. Everybody’s like, ‘I need to wake up.’ They'll shoot up, and you got a couple of minutes, maybe even ten – depending on your body –before you could go out.... You'll see people do that and then smoke or do a line [of cocaine] so you don't go out. But, you feel better. You're not sick now. A lot of people don't want to fall out.*”*

In this sequencing, a person injected fentanyl with xylazine and then smoked or insufflated cocaine, with the functional purpose being to help the person stay awake and alert. This idea of first going “low/down” (i.e., fentanyl/xylazine) before going “high/up” (i.e., crack/cocaine) was mentioned by many participants.

Alternatively, Louis, a 34-year-old White male who has been using opioids for more than a decade, described how he would sequence his drugs when buying from a new seller:

*“*So, I see a new guy, right? I pick up a bag. His shit's got fentanyl and a high amount of xylazine, right? So, the next thing I'm going to do, is I'm going to, on my next shot that I do, I'm going to do his bags, yes, I’m gonna do less bags of it with the xylazine but I'm also going to probably add coke into it to combat that, which I already do, because I'm already lethargic as well from the fentanyl. So I mean, yeah, I would try and add something else to combat it, and that would be what I would use to combat it.*”*

In this way, Louis described a “speedball”—adding cocaine to the injection of fentanyl with xylazine instead of using cocaine afterwards. Louis further expressed that while “speedballing” does help counter the sedative effects of xylazine, he would “rather not do that, if possible.” His cocaine use, therefore, reflected a necessary functional adaptation rather than an active preference to use these drugs together.

Whereas Louis adapted a different polysubstance sequence based on what he expected the embodied sensation to be after use, others created a routine to achieve a particular an embodied sensation over time. For instance, Curtis, a 50-year-old Black male who began using opioids several years ago, described his daily routine:

*“*It goes like this: I wake up, reefer. Boom. After that I wanna fucking smoke crack, base, whatever. I smoke that. Now, I'm jittery. I’m annoyed. [Looking over shoulder] Like ‘who the fuck is that?’ I want to come down. So, I get a bag of dope. Of course, now it has fetty [i.e., fentanyl] in it. So, I do one or two. And I'm still [looking over shoulder] and I'm not high! I'm not feeling balanced yet. I hit that motherfucking tranq with it? I'm feeling like I'm walking on the air.*”*

Curtis’s sequence of substance use reflected decision-making guided by embodied sensation while he is using. Smoking crack/cocaine overstimulated him and the addition of ‘tranq’ (i.e., xylazine) produced the balance Curtis was seeking. This sequencing contrasted with what other participants described, as Curtis was going “high/up” (i.e., crack/cocaine) prior to going “low/down” (i.e., fentanyl/xylazine). In this way, xylazine may offer a perceived utility for addressing symptoms of stimulant “overamping” [48]. Curtis was one of several respondents who endorsed consistently using fentanyl with xylazine last in their daily sequencing of drugs because xylazine is simply too sedating to use any other time. Stated Curtis, definitively:

*“*You can’t do no motherfucking tranq at motherfucking 10 o’ clock in the.

morning. I would be out the whole fucking day.*”*

The perceived utility of using xylazine and crack/cocaine in combination appeared to be bi-directional. Some people preferred to use crack/cocaine before, with, or after xylazine. Irrespective of order or rationale, participants reported drug use patterns that carefully considered the implications of overdose risk and response planning. Their sequential and intentional use may indicate a method of achieving agency over their use patterns.

## Integrative results

**Fig. 3:**
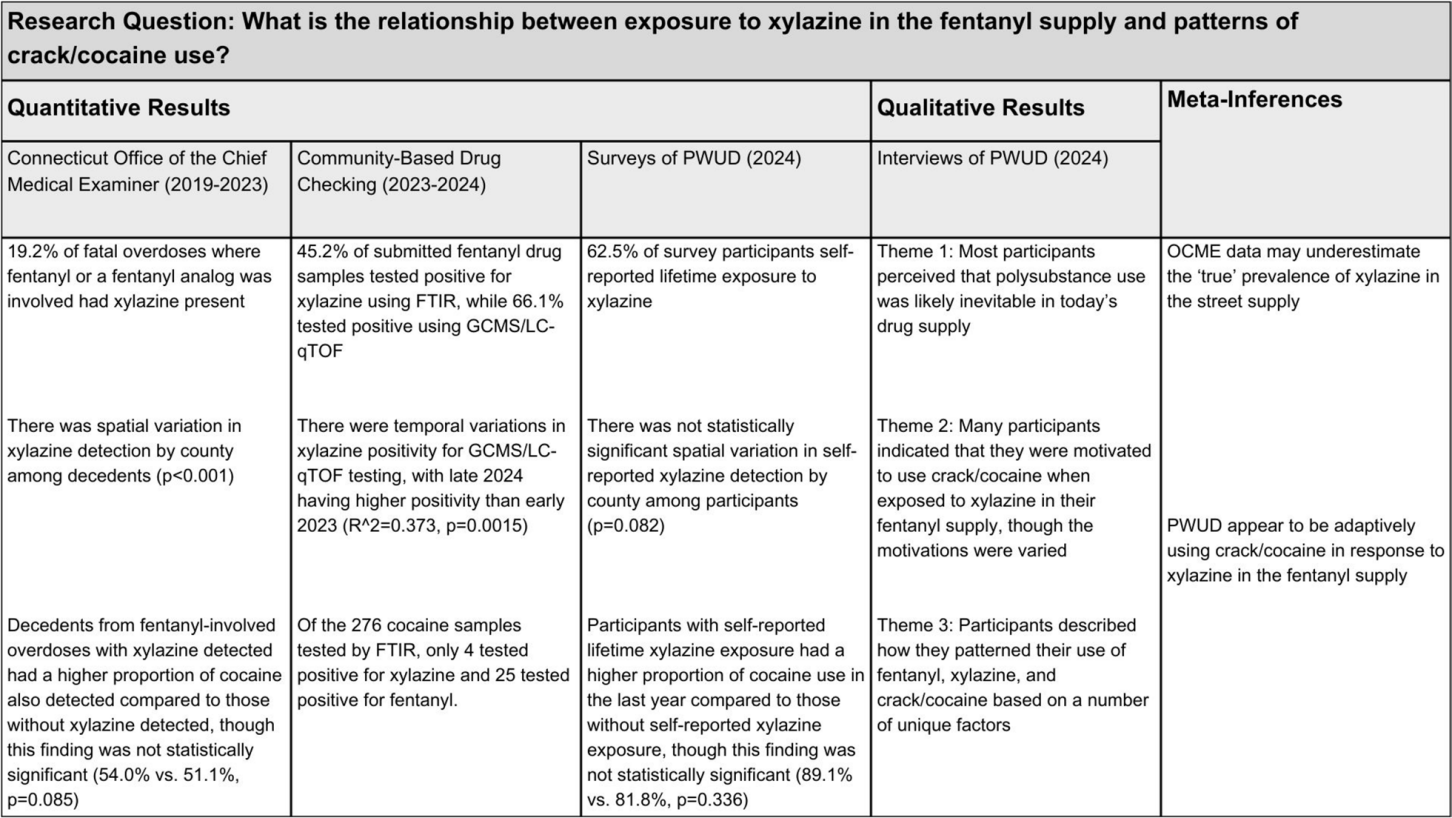
Joint display featuring meta-inferences resulting from mixed methods study exploring cocaine use to manage xylazine exposure in fentanyl in connecticut

Figure 3 provides a joint display of the four unique data sources results alongside the meta-inferences garnered.

### Meta-inference 1: evidence suggests that OCME data underestimates the “true” prevalence of xylazine in the street supply

Quantification of xylazine in CT’s fentanyl supply is difficult to identify for several reasons. Our survey and OCME data demonstrate that xylazine exposure varies temporally and spatially (see Tables 1, 2, and Fig. 2). Trying to confirm such variation using drug checking data has been difficult, as samples were voluntarily provided at limited sites across the state. As yet, there has been no attempt to obtain a representative sample or even a strategic convenience sample. Extrapolating from the limited sample of tests to the experience of individuals, however, our drug checking data suggested that, from 2023–2024, xylazine fluctuated between approximately 45% to 65% of the fentanyl supply (mostly in major to minor components). This is consistent with survey results of those who use fentanyl, with 63% self-reporting inadvertent exposure to xylazine. Further, the qualitative data suggest that participants’ lived experience was that xylazine was either in very little of their fentanyl supply (i.e., ~ 0–10%) or ubiquitous (i.e., ~ 80–100%), perhaps indicating variation by supplier or location. Interestingly, these findings are inconsistent with the OCME data, which demonstrated—on average—25% of those experiencing a fentanyl-involved overdose involved xylazine. We hypothesize—based on integration from our three other data sources—that the OCME data underestimates the “true” prevalence of xylazine in the street drug supply across the state. In the absence of properly scaled community-based drug checking, caution should be exercised in using medical examiner data as a sole litmus test for the prevalence of xylazine in local markets.

### Meta-inference 2: evidence suggest that PWUD are adaptively using crack/cocaine in response to xylazine in the fentanyl supply

We can rule out substantial cross-contamination of fentanyl and crack/cocaine drug supplies. Our community-based drug checking, done via both by FTIR and GCMS/LC-qTOF, indicated that voluntary samples of crack/cocaine tend to have major quantities of cocaine, frequently without fentanyl or xylazine in them; likewise, fentanyl samples tended to be free from large amounts of cocaine. This allows us to establish that PWUD in CT, with the exception of occasional clusters of fatal and non-fatal opioid overdoses in which survivors abjure opioid use, are choosing an individualized approach to polysubstance use. Accordingly, it is likely that sporadic fatalities involving an opioid and cocaine are unlikely to have occurred via cross-contamination of the drug supply. The data from the OCME, that demonstrated the presence of cocaine in fentanyl-involved fatalities—regardless of xylazine detectionhas increased since 2019. While this could suggest that crack/cocaine use has increased over time regardless of xylazine’s presence in the supply, our interview data suggests PWUD are adjusting to xylazine in the local drug supply knowingly. While PWUD cannot always accurately predict when their fentanyl contains xylazine, PWUD may increase crack/cocaine use preemptively, even when xylazine is not present, as a strategy to reduce perceived harms. Further, the qualitative data help contextualize (a) stimulant use as part of PWUDs’ broader pursuit of autonomy and particular embodied sensations while using, and (b) how function during and after use have shaped participants’ reported polysubstance use motivators and patterns.

## Discussion

Our mixed methods study aimed to better understand polysubstance use of stimulants—primarily crack/cocaine—with fentanyl and xylazine among PWUD in CT. Both OCME data from 2019 to 2023 and survey data collected from PWUD in 2024 indicated that crack/cocaine use was more common among those exposed to xylazine than those without xylazine exposure. Though neither of these quantitative findings were statistically significant, we believe that the similarity of the results in the two independent data sources suggest a possibility that people are using crack/cocaine in response to the increasing prevalence of xylazine in the fentanyl supply. This possibility is worthy of further investigation. Further, our qualitative interviews with PWUD in 2024 provided evidence that this increased use is part of a conscious strategy to (a) manage xylazine sedation by creating a balance between “uppers” and “downers”, (b) regain or maintain daily functioning, (c) maintain a sense of safety, and even (d) practice harm reduction (e.g., perceived overdose prevention, attention to XROs, and other aspects of situational well-being).

These findings are well situated within the current research landscape, which suggests that those who use opioids, are exposed to xylazine in their supply and experience intense, unintended sedation, have an increased risk of negative outcomes such as robbery, assault, or accidents [49]. Logic suggests that PWUD under these circumstances may seek adaptive means to self-manage and prevent such negative effects. Further, our survey data is similar to published literature, insofar as it suggests that those who are exposed to xylazine are more likely to have recent polysubstance use [50–52]. Additionally, those who only use stimulants have been shown to have little knowledge of xylazine, [53] indicating that the polysubstance triad of opioid-stimulant-sedative may potentially only be known to those with prior experience of opioids adulterated with sedatives.

## Findings from data integration

We faced two primary challenges in the pursuit of studying the current drug supply and polysubstance use in the context of it. First, the “truths” of our nation’s volatile drug supply are oversimplified. Drug markets are rapidly changing on spatial and temporal scales that even experts do not currently understand. Second, any single data source in this field is insufficient to capture the complexities of such a supply. Thus, the place for mixed methods research in the study of the drug supply and how people are using drugs in this supply is evident.

### Justification for meta-inference 1: OCME data and xylazine prevalence

Based on the results of this study, we suggest that OCME data is likely an underestimate of the “true” xylazine prevalence in the street supply. We propose some potential mechanisms to account for this. The first possible mechanism is behavioral: xylazine’s presence in the fentanyl supply may drive people to use in ways that are protective against overdose (e.g., use less, use slower, not use alone, etc.). Our qualitative data lend credence to this possibility. Hence, the OCME data may represent a portion of PWUD who are unaware of these potential harm reduction strategies and therefore at higher risk for a fatal overdose. The second potential mechanism is structural in nature: those experiencing an overdose may be uniquely different from that of a population that has survived. For instance, recently in Connecticut, the proportion of older Black men among the decedents has been increasing, perhaps as a consequence of their environments that induce respiratory problems that can increase the risk of respiratory failure following fentanyl exposure [54–57]. While this subpopulation may be at higher risk, other subpopulations may be at lower risk. The third potential mechanism is provisional: xylazine’s presence in the supply may, in some ways, be protective [23]. The sedation produced by the addition of xylazine can mask the rapidly waning narcotic effect of a lower amount of fentanyl in each package. Less fentanyl is less likely to cause fatal respiratory depression and overdose than the less potent xylazine that has replaced it. A fourth possible mechanism is physiological: xylazine competes with fentanyl for binding to opioid receptors in the locus coeruleus, the site in the brain involved in respiratory rate [58, 59]. This might lower the effective dose of fentanyl, and hence the threat of fatal overdose. The fifth and final potential mechanism is the idea presented herein: that xylazine’s presence in the fentanyl supply may increase use of crack/cocaine in ways that are adaptive to survival. None of these mechanisms is mutually exclusive.

### Justification for meta-inference 2: adaptive crack/cocaine use among PWUD

Our study additionally supports the finding that PWUD are increasing their use of crack/cocaine adaptively in response to xylazine in their fentanyl supply. In examining polysubstance use, it is important to parse out the relative consumption patterning, timing, motivations, and whether the use of is intentional or unintentional. Our study adds to the literature on concurrent use of stimulants and fentanyl (3), as PWUD have begun to change certain patterns, timing, and motivations in response to xylazine exposure. There are likely unknown long-term health impacts associated with the use of an opioid-stimulant-sedative triad. While xylazine produces both α-adrenergic receptor agonist sedation and κ-opioid receptor agonist action [14, 16, 59], the mechanism by which cocaine alters physiological responses during polysubstance use and individuals’ subjective experiences remain unclear.

While crack/cocaine is the dominant stimulant in CT, methamphetamine use dominates elsewhere in the US [60]. The concurrent use of methamphetamines and fentanyl is well established [61–63], but there is limited information on cross-contamination of methamphetamine and xylazine in the drug supply, nor is there much published on the physiological responses or psychological adaptations of people exposed to the three drugs [64, 65]. The approach we describe in conducting our study might be useful to researchers studying the polysubstance use of fentanyl, xylazine, and methamphetamine.

## Future research

Since clinical studies of xylazine in humans cannot be conducted for ethical and regulatory reasons, greater effort should be directed to obtaining more qualitative data from individuals who are exposed to xylazine either alone or in combination with fentanyl and/or cocaine. It might also be possible to organize field studies in which community-based drug checking is combined with post-use qualitative interviewing to disentangle the effects experienced by people who are exposed to xylazine alone or in combination. Such studies would generate actionable evidence to aid public health practitioners, policymakers, clinicians, and most importantly PWUD, to help inform tools and interventions which can prevent immediate physiological harms, including overdose, overamping, and excessive sedation. Ultimately, there are structural approaches to managing the uncertainties of the current US drug supply and its deleterious consequences. For instance, overdose prevention centers and safer supply programs, which remain illegal in many places nationally due to punitive and archaic policies, are evidence-based harm reduction strategies that would complement individual-level approaches to reversing the opioid overdose crisis [66–68].

One direction for future research is predicated on the evidence across New England that methamphetamine use might be becoming increasingly popular in the region [69, 70]. Understanding if, and how, this is influenced by xylazine or other non-human sedatives (e.g., medetomidine) that have become increasingly common in the regional supply could reveal other patterns about polysubstance use related to opioid-sedative-stimulant interactions not revealed here. Conversely, areas with high levels of methamphetamine use may consider if and how such use is impacted by sedatives such as xylazine or medetomidine in their supply.

Research related to polysubstance use involving opioids, sedatives, and stimulants could explore key variables that appeared as statistically significant in our bivariate analyses in the OCME data or the survey data. Specifically, it may be important to explore how spatial variation, age, gender, housing status, race, and/or ethnicity play a role in patterning around decisions about crack/cocaine use in the context of fentanyl with xylazine in the drug supply. For instance, our qualitative data—though we had a small number of female participants—suggests that women, especially those who are unhoused, may have extra motivation to use crack/cocaine when exposed to xylazine in order to stay safe in the streets (e.g., to ensure their items are not stolen, to stay awake at night, etc.). More research is needed to understand the reasons for these patterns and others, and how people learn to modify their behaviors to protect themselves.

Our study revealed that adaptive crack/cocaine use allowed PWUD to maintain a self-evaluated sufficient and appropriate level of functioning in social settings. Understanding how polysubstance use may impact PWUD in spheres such as employment, housing, and social connection will become increasingly important if sedatives continue to remain prevalent in the unregulated fentanyl supply. The emergence of sedatives that are stronger than xylazine (e.g., medetomidine), additionally sedating (designer benzodiazepines), and opioids stronger than fentanyl (e.g., isotonitazine) may lead PWUD to increasingly turn to stimulants in an attempt to manage the embodied effects they are experiencing. Researchers should be prepared to study, and clinical and harm reduction providers should be prepared to help PWUD reduce their risks, when such novel substances become a regular part of polysubstance use. Equipped with the tools of data integration, we can begin to create localized approximations of what is happening in almost real time such that the findings may prove meaningful to researchers, clinicians, policymakers, and, most important, PWUD themselves.

## Limitations

As with any mixed methods study, each dataset and method presented limitations; the most salient are described here. Our community-based drug checking data came from voluntary samples of drug remnants—which can be unknowingly cross-contaminated with multiple substances—from four different testing locations. The fluctuating numbers of samples in each locale over time **(see **Table 1** and **Fig. 1**, Panel E)** were not likely a true indication of the sales volumes. More broadly, community-based drug checking (in contrast to event-based drug checking) is still in its infancy in this country, and testing sites and researchers must be certain not to make sweeping claims that such data are representative of entire communities or spatial locations. We are largely unaware of (a) the choices people make when volunteering samples, (b) the spatial distribution that such samples represent, and (c) if samples always represent the consumed drug or some contaminated form. Thus, drug checking data must be interpreted with caution and their generalizability is limited. Further, drug checking technology is not without limitations (e.g., varying sensitivity, cross-reactivity, limits of detection, technician error, limited spectra libraries, etc.) [35, 71, 72], so results must be interpreted with caution.

Both the survey and qualitative datasets were based on self-report, limiting our ability to confirm what substances participants had been exposed to amidst a volatile drug supply. Many participants were engaged with harm reduction organizations in drug checking or at methadone programs that used urine screening, increasing the confidence in our findings; yet it is important to consider that sampling bias that may have resulted from recruiting a convenience sample of PWUD from harm reduction organizations and those utilizing drug checking services. Lastly, while the Connecticut OCME overdose mortality data provides a large sample size, our survey data was built from a convenience sample of a small number of PWUD in Connecticut and may be insufficiently powered to detect statistically significant differences between groups should they exist.

## Conclusions

Our study demonstrates that a mixed method approach is appropriate and necessary to improve our understanding of local drug markets. By integrating four datasets, we have demonstrated that PWUD in Connecticut are intentionally increasing their crack/cocaine use, potentially to manage xylazine exposure in their fentanyl supply. Additionally, the results suggest that reliance upon medical examiner data—particularly in areas of the country that lack drug checking services—may underestimate the prevalence of xylazine in local, street supplies.

## Data Availability

Connecticut overdose records are available, in part, online through Connecticut Open Data. Connecticut community-based drug checking data, in part, can be seen online using STASH ID. The interview and survey data involved in this secondary analysis are not publicly available.

## References

[1] Tanz LJ, Miller KD, Dinwiddie AT, Gladden RM, Asher A, Baldwin G, et al. Drug overdose deaths involving stimulants ― United States, January 2018–June 2024. MMWR Morb Mortal Wkly Rep. 2025;74(32):491–9. 10.15585/mmwr.mm7432a1. 40875496 10.15585/mmwr.mm7432a1 PMC12393691

[2] Lundstrom EW. Synthetic opioid and stimulant co-involved overdose deaths by occupation and industry — United States, 2022. MMWR Morb Mortal Wkly Rep. 2025. 10.15585/mmwr.mm7410a3. 40146666 10.15585/mmwr.mm7410a3 PMC11949317

[3] Ciccarone D. The rise of illicit fentanyls, stimulants and the fourth wave of the opioid overdose crisis. Curr Opin Psychiatry. 2021;34(4):344–50. 10.1097/YCO.0000000000000717. 33965972 10.1097/YCO.0000000000000717 PMC8154745

[4] Font-Mayolas S, Calvo F. Polydrug definition and assessment: the state of the art. IJERPH. 2022;19(20):13542. 10.3390/ijerph192013542. 36294127 10.3390/ijerph192013542 PMC9602920

[5] Shearer RD, Bart G, Reznikoff C. Rethinking the use of “polysubstance” to describe complex substance use patterns. J Gen Intern Med. 2022;37(12):3174–5. 10.1007/s11606-022-07424-5. 35112282 10.1007/s11606-022-07424-5 PMC9485401

[6] Bunting AM, Shearer R, Linden-Carmichael AN, Williams AR, Comer SD, Cerdá M, et al. Are you thinking what I’m thinking? Defining what we mean by “polysubstance use.” Am J Drug Alcohol Abuse. 2024;50(1):1–7. 10.1080/00952990.2023.2248360. 37734160 10.1080/00952990.2023.2248360 PMC10939915

[7] Xu KY, Lin L, Grucza RA. Polysubstance use is not synonymous with poly substance use disorder. Am J Public Health. 2025;115(5):646–8. 10.2105/AJPH.2025.308070. 40203259 10.2105/AJPH.2025.308070 PMC11983058

[8] Tanz LJ. Detection of illegally manufactured fentanyls and carfentanil in drug overdose deaths — United States, 2021–2024. MMWR Morb Mortal Wkly Rep. 2024. 10.15585/mmwr.mm7348a2. 39636782 10.15585/mmwr.mm7348a2 PMC11620336

[9] Pergolizzi J Jr, Raffa R, LeQuang JAK, Breve F, Varrassi G. Old drugs and new challenges: a narrative review of nitazenes. Cureus. 2023. 10.7759/cureus.40736. 37485167 10.7759/cureus.40736 PMC10361140

[10] Gladden RM, O’Donnell J, Mattson CL, Seth P. Changes in opioid-involved overdose deaths by opioid type and presence of benzodiazepines, cocaine, and methamphetamine — 25 states, July–December 2017 to January–June 2018. MMWR Morb Mortal Wkly Rep. 2019;68(34):737–44. 10.15585/mmwr.mm6834a2. 31465320 10.15585/mmwr.mm6834a2 PMC6715260

[11] Valente PK, Bazzi AR, Childs E, Salhaney P, Earlywine J, Olson J, et al. Patterns, contexts, and motivations for polysubstance use among people who inject drugs in non-urban settings in the U.S. Northeast. Int J Drug Policy. 2020;85:102934. 10.1016/j.drugpo.2020.102934. 32911318 10.1016/j.drugpo.2020.102934 PMC7770041

[12] Kariisa M, O’Donnell J, Kumar S, Mattson CL, Goldberger BA. Illicitly manufactured fentanyl–involved overdose deaths with detected Xylazine — United States, January 2019–June 2022. MMWR Morb Mortal Wkly Rep. 2023;72(26):721–7. 10.15585/mmwr.mm7226a4. 37384558 10.15585/mmwr.mm7226a4 PMC10328484

[13] Huo S. Notes from the field: suspected medetomidine withdrawal syndrome among fentanyl-exposed patients — Philadelphia, Pennsylvania, September 2024–January 2025. MMWR Morb Mortal Wkly Rep. 2025. 10.15585/mmwr.mm7415a2. 40310762 10.15585/mmwr.mm7415a2 PMC12045483

[14] Kacinko SL, Mohr ALA, Logan BK, Barbieri EJ. Xylazine: pharmacology review and prevalence and drug combinations in forensic toxicology casework. J Anal Toxicol. 2022;46(8):911–7. 10.1093/jat/bkac049. 35770859 10.1093/jat/bkac049

[15] Alexander RS, Canver BR, Sue KL, Morford KL. Xylazine and overdoses: trends, concerns, and recommendations. Am J Public Health. 2022;112(8):1212–6. 10.2105/AJPH.2022.306881. 35830662 10.2105/AJPH.2022.306881 PMC9342814

[16] Ćupić V, Čolić M, Pavičić L, Vučević D, Varagić VM. Immunomodulatory effect of xylazine, an α2 adrenergic agonist, on rat spleen cells in culture. J Neuroimmunol. 2001;113(1):19–29. 10.1016/S0165-5728(00)00370-2. 11137573 10.1016/s0165-5728(00)00370-2

[17] Samanta A, Roffe C, Woods KL. Accidental self administration of xylazine in a veterinary nurse. Postgrad Med J. 1990;66(773):244–5. 10.1136/pgmj.66.773.244. 2362897 10.1136/pgmj.66.773.244 PMC2429459

[18] T.Kumaravadivel D, Ganapathy GK, Mugunthan M. S. accidental self-injection of xylazine during work: A rare case [Internet]. 2018 Jul. Available from: https://www.researchgate.net/publication/326129001_ACCIDENTAL_SELF-INJECTION_OF_XYLAZINE_DURING_WORK_A_RARE_CASE

[19] Capraro AJ, Wiley JF, Tucker JR. Severe intoxication from xylazine inhalation. Pediatr Emerg Care. 2001;17(6):447–8. 10.1097/00006565-200112000-00012. 11753193 10.1097/00006565-200112000-00012

[20] Ball NS, Knable BM, Relich TA, Smathers AN, Gionfriddo MR, Nemecek BD, et al. Xylazine poisoning: a systematic review. Clin Toxicol Phila. 2022;60(8):892–901. 10.1080/15563650.2022.2063135. 35442125 10.1080/15563650.2022.2063135

[21] Ayub S, Parnia S, Poddar K, Bachu AK, Sullivan A, Khan AM, et al. Xylazine in the opioid epidemic: a systematic review of case reports and clinical implications. Cureus. 2023. 10.7759/cureus.36864. 37009344 10.7759/cureus.36864 PMC10063250

[22] Choi S, Irwin MR, Kiyatkin EA. Xylazine effects on opioid-induced brain hypoxia. Psychopharmacol Berl. 2023;240(7):1561–71. 10.1007/s00213-023-06390-y. 10.1007/s00213-023-06390-y PMC10775769 37340247

[23] Love JS, Levine M, Aldy K, Brent J, Krotulski AJ, Logan BK, et al. Opioid overdoses involving xylazine in emergency department patients: a multicenter study. Clin Toxicol Phila. 2023;61(3):173–80. 10.1080/15563650.2022.2159427. 37014353 10.1080/15563650.2022.2159427 PMC10074294

[24] Zagorski CM, Hosey RA, Moraff C, Ferguson A, Figgatt M, Aronowitz S, et al. Reducing the harms of xylazine: clinical approaches, research deficits, and public health context. Harm Reduct J. 2023;20(1):141. 10.1186/s12954-023-00879-7. 37777769 10.1186/s12954-023-00879-7 PMC10544173

[25] Hill K, Minahan-Rowley R, Biegacki ET, Heimer R, Sue KL. Providers’ knowledge and perception of xylazine in the unregulated drug supply: a sequential explanatory mixed-methods study. Harm Reduct J. 2024;21(1):148. 10.1186/s12954-024-01052-4. 39148036 10.1186/s12954-024-01052-4 PMC11328386

[26] Dowton A, Doernberg M, Heiman E, Barelli P, Golden M, Wang H, et al. Recognition and treatment of wounds in persons using xylazine: a case report from New Haven, Connecticut. J Addict Med. 2023. 10.1097/ADM.0000000000001198. 37934550 10.1097/ADM.0000000000001198

[27] Sue KL, Hawk K. Clinical considerations for the management of xylazine overdoses and xylazine‐related wounds. Addict. 2023. 10.1111/add.16388. 10.1111/add.16388 37939387

[28] Rhee TG, Ross JS, Rosenheck RA, Grau LE, Fiellin DA, Becker WC. Accidental drug overdose deaths in Connecticut, 2012–2018: the rise of polysubstance detection? Drug Alcohol Depend. 2019;205:107671. 10.1016/j.drugalcdep.2019.107671. 31706248 10.1016/j.drugalcdep.2019.107671

[29] Thangada S, Clinton H, Ali S. Notes from the field: xylazine, a veterinary tranquilizer, identified as an emerging novel substance in drug overdose deaths — Connecticut, 2019–2020. MMWR Morb Mortal Wkly Rep. 2021;70:1303–4. 10.15585/mmwr.mm7037a5. 34529638 10.15585/mmwr.mm7037a5 PMC8445375

[30] CT DPH. Unintentional drug overdose deaths in connecticut: a fact sheet – 2020 update [Internet]. 2020 Mar. Report. Available from: https://portal.ct.gov/-/media/dph/injury-and-violence-prevention/opioid-overdose-data/fact-sheets/fact-sheet-unintentional-drug-overdose-deaths_2019_4_24_2020.pdf?rev=8a4730cfd4604979b45cadff4ea74006&hash=594CAB41788CBFCB1E86E281C8C45331

[31] Nunez J, DeJoseph ME, Gill JR. Xylazine, a veterinary tranquilizer, detected in 42 accidental Fentanyl intoxication deaths. Am J Forensic Med Pathol. 2021;42(1):9–11. 10.1097/PAF.0000000000000622. 33031124 10.1097/PAF.0000000000000622

[32] Hill K, Heimer R, Sue KL. “Everybody’s liking it”—intentional use of Xylazine in Fentanyl among people who use drugs. Subst Use Misuse. 2025. 10.1080/10826084.2025.2549499. 40847818 10.1080/10826084.2025.2549499

[33] Bluthenthal RN, Malik MR, Grau LE, Singer M, Marshall P, Heimer R. Sterile syringe access conditions and variations in HIV risk among drug injectors in three cities. Addict. 2004;99(9):1136–46. 10.1111/j.1360-0443.2004.00694.x. 10.1111/j.1360-0443.2004.00694.x 15317634

[34] Heimer R, Barbour R, Palacios WR, Nichols LG, Grau LE. Associations between injection risk and community disadvantage among suburban injection drug users in Southwestern Connecticut, USA. AIDS Behav. 2014;18(3):452–63. 10.1007/s10461-013-0572-3. 23921583 10.1007/s10461-013-0572-3 PMC3917972

[35] Green TC, Park JN, Gilbert M, McKenzie M, Struth E, Lucas R, et al. An assessment of the limits of detection, sensitivity and specificity of three devices for public health-based drug checking of Fentanyl in street-acquired samples. Int J Drug Policy. 2020;77:102661. 10.1016/j.drugpo.2020.102661. 31951925 10.1016/j.drugpo.2020.102661

[36] Ujeneza M, Tardif J, Thompson E, Badea A, Morales A, Altomare‐Jarczyk C, et al. Point‐of‐care drug‐checking: assessing the Rhode Island drug supply using FTIR spectroscopy to detect Fentanyl, Xylazine and other substances. Drug Alcohol Rev. 2025;44(7):1866–74. 10.1111/dar.70037. 40977066 10.1111/dar.70037 PMC12947298

[37] Green TC, Olson R, Jarczyk C, Erowid E, Erowid F, Thyssen S, et al. Implementation and uptake of the Massachusetts drug supply data stream: a statewide public health-public safety partnership drug checking program. J Public Health Manag Pract. 2022;28(Supplement 6):S347–54. 10.1097/PHH.0000000000001581. 36194805 10.1097/PHH.0000000000001581 PMC9531987

[38] Uljon S. Advances in fentanyl testing. In: Advances in clinical chemistry [Internet]. Elsevier; 2023 [cited 2025 Aug 21]. p. 1–30. Available from: https://linkinghub.elsevier.com/retrieve/pii/S006524232300049510.1016/bs.acc.2023.05.004 10.1016/bs.acc.2023.05.004 37852717

[39] Norman C, Marland V, McKenzie C, Ménard H, Nic Daéid N. Evaluation of Fentanyl immunoassay test strips for rapid in-situ detection of Fentanyl and Fentanyl analogs in seized samples and alternative matrices. Int J Drug Policy. 2023;118:104102. 10.1016/j.drugpo.2023.104102. 37343365 10.1016/j.drugpo.2023.104102

[40] Collins AB, Wightman RS, Macon EC, Guan Y, Shihipar A, Krieger M, et al. Comprehensive testing and rapid dissemination of local drug supply surveillance data in Rhode Island. Int J Drug Policy. 2023;118:104118. 10.1016/j.drugpo.2023.104118. 37422985 10.1016/j.drugpo.2023.104118

[41] Saunders B, Sim J, Kingstone T, Baker S, Waterfield J, Bartlam B, et al. Saturation in qualitative research: exploring its conceptualization and operationalization. Qual Quant. 2018;52(4):1893–907. 10.1007/s11135-017-0574-8. 29937585 10.1007/s11135-017-0574-8 PMC5993836

[42] Braun V, Clarke V. Using thematic analysis in psychology. Qual Res Psychol. 2006;3(2):77–101. 10.1191/1478088706qp063oa.

[43] Fetters MD, Curry LA, Creswell JW. Achieving integration in mixed methods designs—Principles and practices. Health Serv Res. 2013;48(6pt2):2134–56. 10.1111/1475-6773.12117. 24279835 10.1111/1475-6773.12117 PMC4097839

[44] Younas A, Fàbregues S, Munce S, Creswell JW. Framework for types of metainferences in mixed methods research. BMC Med Res Methodol. 2025;25(1):18. 10.1186/s12874-025-02475-8. 39856568 10.1186/s12874-025-02475-8 PMC11758751

[45] Guetterman TC, Manojlovich M. Grand rounds in methodology: designing for integration in mixed methods research. BMJ Qual Saf. 2024;33(7):470–8. 10.1136/bmjqs-2023-016112. 38575310 10.1136/bmjqs-2023-016112

[46] Kral AH, Lambdin BH, Browne EN, Wenger LD, Bluthenthal RN, Zibbell JE, et al. Transition from injecting opioids to smoking fentanyl in San Francisco, California. Drug Alcohol Depend. 2021;227:109003. 10.1016/j.drugalcdep.2021.109003. 34482046 10.1016/j.drugalcdep.2021.109003 PMC10790652

[47] Tanz LJ, Gladden RM, Dinwiddie AT, Miller KD, Broz D, Spector E, et al. Routes of drug use among drug overdose deaths — United States, 2020–2022. MMWR Morb Mortal Wkly Rep. 2024;73(6):124–30. 10.15585/mmwr.mm7306a2. 38358969 10.15585/mmwr.mm7306a2 PMC10899081

[48] National harm reduction coalition. What is overamping? [Internet]. 2024 [cited 2025 Sep 11]. Available from: https://harmreduction.org/issues/overdose-prevention/overview/stimulant-overamping-basics/what-is-overamping/

[49] Reed MK, Martin González K, Green TC, Esteves Camacho T, Laurano R, Clark-García F, et al. “Anything can happen”: experiences of people using opioids in a xylazine market. Harm Reduct J. 2025;22(1):134. 10.1186/s12954-025-01275-z. 40745293 10.1186/s12954-025-01275-z PMC12312578

[50] Tan M, Nassau T, Kuncio D, Higgins D, Teixeira Da Silva D, Tomlinson D, et al. Xylazine use among people who inject drugs, Philadelphia 2022. J Addict Med. 2024;18(2):194–200. 10.1097/ADM.0000000000001264. 38289240 10.1097/ADM.0000000000001264

[51] Jiang X, Connolly S, Strahan AE, Rivera Blanco L, Mikosz CA, Guy GP, et al. Reported xylazine use among adults aged ≥18 years evaluated for substance use treatment — United States, July 2022–September 2023. MMWR Morb Mortal Wkly Rep. 2024;73(26):594–9. 10.15585/mmwr.mm7326a2. 38959171 10.15585/mmwr.mm7326a2 PMC11221633

[52] Rudolph JE, Martinez-Ocasio LM, Feder KA, Tomko C, Cepeda JA, Kirk GD, et al. Characterizing patterns of opioid and stimulant use by route and associations with non-fatal overdose and xylazine use in people who have injected drugs from Baltimore, MD, 2023–2024. Drug Alcohol Depend Rep. 2026;18:100403. 10.1016/j.dadr.2025.100403. 41488161 10.1016/j.dadr.2025.100403 PMC12757556

[53] Kelly PJA, Green TC, Rich JD, Vento SA, Bailey A, Silva V, et al. Xylazine awareness and suspected presence in the illicit drug supply among people who used stimulants in an overdose hotspot, 2023. Subst Use Misuse. 2026;61(2):217–26. 10.1080/10826084.2025.2549501. 40888200 10.1080/10826084.2025.2549501 PMC12826337

[54] Bazargan M, Smith JL, Robinson P, Uyanne J, Abdulrahoof R, Chuku C, et al. Chronic respiratory disease and health-related quality of life of African American older adults in an economically disadvantaged area of Los Angeles. Int J Environ Res Public Health. 2019;16(10):1756. 10.3390/ijerph16101756. 31108963 10.3390/ijerph16101756 PMC6571607

[55] Schuyler AJ, Wenzel SE. Historical redlining impacts contemporary environmental and asthma-related outcomes in Black adults. Am J Respir Crit Care Med. 2022;206(7):824–37. 10.1164/rccm.202112-2707OC. 35612914 10.1164/rccm.202112-2707OC PMC9799280

[56] Lee EK, Donley G, Ciesielski TH, Gill I, Yamoah O, Roche A, et al. Health outcomes in redlined versus non-redlined neighborhoods: a systematic review and meta-analysis. Soc Sci Med. 2022;294:114696. 10.1016/j.socscimed.2021.114696. 34995988 10.1016/j.socscimed.2021.114696

[57] Mein SA, Liu M, Marinacci LX, Rice MB, Wadhera RK. Neighborhood exposome and prevalence of asthma and Chronic Obstructive Pulmonary Disease in the United States. Ann Am Thorac Soc. 2025;22(5):797–801. 10.1513/AnnalsATS.202409-991RL. 39928483 10.1513/AnnalsATS.202409-991RL PMC12051918

[58] Floresta G, Granzotto A, Patamia V, Arillotta D, Papanti GD, Guirguis A, et al. Xylazine as an emerging new psychoactive substance; focuses on both 5‐HT_7_ and κ‐opioid receptors’ molecular interactions and isosteric replacement. Arch Pharm (Weinheim). 2025;358(3):e2500041. 10.1002/ardp.202500041. 40091602 10.1002/ardp.202500041 PMC11911908

[59] Bedard ML, Murray JG, Huang XP, Nowlan AC, Conley SY, Mott SE, et al. Xylazine is an agonist at kappa opioid receptors and exhibits sex-specific responses to naloxone administration [preprint] [Internet]. Neuroscience; 2023 Sep [cited 2023 Oct 3]. Report. Available from: http://biorxiv.org/lookup/doi/10.1101/2023.09.08.556914

[60] Jones CM, Houry D, Han B, Baldwin G, Vivolo‐Kantor A, Compton WM. Methamphetamine use in the United States: epidemiological update and implications for prevention, treatment, and harm reduction. Ann N Y Acad Sci. 2022;1508(1):3–22. 10.1111/nyas.14688. 34561865 10.1111/nyas.14688 PMC9097961

[61] Rawson RA, Erath TG, Clark HW. The fourth wave of the overdose crisis: examining the prominent role of psychomotor stimulants with and without fentanyl. Prev Med. 2023;176:107625. 10.1016/j.ypmed.2023.107625. 37468073 10.1016/j.ypmed.2023.107625

[62] Strickland JC, Havens JR, Stoops WW. A nationally representative analysis of “twin epidemics”: rising rates of methamphetamine use among persons who use opioids. Drug Alcohol Depend. 2019;204:107592. 10.1016/j.drugalcdep.2019.107592. 31586804 10.1016/j.drugalcdep.2019.107592 PMC6884137

[63] Al-Tayyib A, Koester S, Langegger S, Raville L. Heroin and methamphetamine injection: an emerging drug use pattern. Subst Use Misuse. 2017;52(8):1051–8. 10.1080/10826084.2016.1271432. 28323507 10.1080/10826084.2016.1271432 PMC5642954

[64] Wagner KD, Fiuty P, Page K, Tracy EC, Nocera M, Miller CW, et al. Prevalence of fentanyl in methamphetamine and cocaine samples collected by community-based drug checking services. Drug Alcohol Depend. 2023;252:110985. 10.1016/j.drugalcdep.2023.110985. 37826988 10.1016/j.drugalcdep.2023.110985 PMC10688611

[65] Ivsins A, Fleming T, Barker A, Mansoor M, Thakarar K, Sue K, et al. The practice and embodiment of “goofballs”: a qualitative study exploring the co-injection of methamphetamines and opioids. Int J Drug Policy. 2022;107:103791. 10.1016/j.drugpo.2022.103791. 35830749 10.1016/j.drugpo.2022.103791 PMC10894463

[66] Dunham K, Hill K, Kazal H, Butner JL, Hull I, Sue K, et al. In support of Overdose Prevention Centers: position statement of AMERSA, Inc (Association for Multidisciplinary Education and Research in Substance Use and Addiction). Subst Use &amp; Addict J. 2024. 10.1177/29767342241252590. 10.1177/29767342241252590 38747578

[67] Ledlie S, Garg R, Cheng C, Kolla G, Antoniou T, Bouck Z, et al. Prescribed safer opioid supply: a scoping review of the evidence. Int J Drug Policy. 2024;125:104339. 10.1016/j.drugpo.2024.104339. 38335867 10.1016/j.drugpo.2024.104339

[68] Haines M, O’Byrne P. Safer Stimulant Supply: program outcomes. Can J Nurs Res. 2025;57(2):324–9. 10.1177/08445621251314227. 39865880 10.1177/08445621251314227 PMC12086283

[69] Streck JM, Klevens RM, O’Cleirigh C, Batchelder AW. Injection of methamphetamine has increased in Boston, Massachusetts: 5 waves of Centers for Disease Control and Prevention state surveillance data. J Addict Med. 2023;17(3):349–52. 10.1097/ADM.0000000000001107. 37267188 10.1097/ADM.0000000000001107 PMC10149569

[70] Hoffman J. As Fentanyl Deaths Slow, Meth Comes for Maine [Internet]. 2025. Available from: https://www.nytimes.com/2025/04/16/health/meth-maine-fentanyl.html

[71] Harper L, Powell J, Pijl EM. An overview of forensic drug testing methods and their suitability for harm reduction point-of-care services. Harm Reduct J. 2017;14(1):52. 10.1186/s12954-017-0179-5. 28760153 10.1186/s12954-017-0179-5 PMC5537996

[72] Carroll JJ, Mackin S, Schmidt C, McKenzie M, Green TC. The Bronze Age of drug checking: barriers and facilitators to implementing advanced drug checking amidst police violence and COVID-19. Harm Reduct J. 2022;19(1):9. 10.1186/s12954-022-00590-z. 35120531 10.1186/s12954-022-00590-z PMC8814788

